# Lime and Fly Ash Co-Solidification Treatment of Oil-Contaminated Soil: Characteristics in Different Water Environments and Evaluation of Engineering Reuse

**DOI:** 10.3390/toxics14050357

**Published:** 2026-04-23

**Authors:** Hemiao Yu, Pei Gao, Hui Li, Min Li

**Affiliations:** 1 College of Civil Engineering and Transportation, Hebei University of Technology, Tianjin 300401, China; 2 Key Laboratory for Green Chemical Technology of Ministry of Education, School of Chemical Engineering and Technology, Tianjin University, Tianjin 300072, China; 3 Ningbo Key Laboratory of Green Petrochemical Carbon Emission Reduction Technology and Equipment, Zhejiang Institute of Tianjin University, Ningbo 315201, China

**Keywords:** oil-contaminated soil, solidification stabilization, lime and fly ash, migration and diffusion, permeability, engineering reuse

## Abstract

Stabilization/solidification (S/S) is a crucial technology for the engineering reuse of oil-contaminated soil. A key challenge, however, is preventing the migration of residual oil under varying hydraulic conditions. This study investigates the efficacy of a lime and fly ash binder in treating oil-contaminated soil. We systematically compared the performance of untreated (UOCS) and treated (TOCS) soils under different aqueous environments (humidity injection, water injection, and permeation). We evaluated oil migration, water-holding capacity, and permeability characteristics. The results demonstrate that the lime–fly ash treatment effectively adsorbed and immobilized oil contaminants, restricting their mobility to a remarkably low range of 0.54% to 4.90%. Furthermore, the S/S treatment significantly improved the soil’s hydraulic properties: it enhanced the water-holding capacity, reduced the soil-water characteristic curve hysteresis, and counteracted the oil-induced hydrophobicity. Consequently, the effective permeation channels were restored, leading to a higher permeability coefficient in TOCS compared to UOCS. Crucially, the hydro-mechanical performance of the treated soil met the criteria of the Solidification/Stabilization Resource Guide, confirming its suitability for engineering applications.

## 1. Introduction

Oil contamination of soil, a widespread consequence of petroleum extraction, transportation, and processing, poses a significant environmental and geotechnical challenge [1,2]. The presence of oil, a complex mixture of toxic and persistent hydrocarbons [3], fundamentally alters soil properties. These hydrophobic compounds tend to adsorb onto soil particles or occupy pore spaces [4,5], which not only impairs soil structure and permeability but also triggers changes in wettability and other critical physicochemical characteristics [6]. This degradation of soil quality often renders it unsuitable for conventional use and raises concerns about the migration and diffusion of contaminants into the surrounding environment [7,8,9,10,11].

To address this issue, stabilization/solidification (S/S) has emerged as a promising approach for converting oil-contaminated soil from hazardous waste into a viable geotechnical construction material [12]. Among various solidified materials, the combination of lime and fly ash is particularly attractive. Lime, a widely available and cost-effective inorganic material, undergoes pozzolanic reactions with aluminosilicates in the soil [13], forming cementitious products such as calcium silicate hydrate (C-S-H) and calcium aluminate hydrate (C-A-H), which substantially improve the mechanical strength of the treated soil [14]. Fly ash, a major industrial by-product from coal-fired power plants, has a global annual production of several hundred million tons [15]; its massive stockpiling not only occupies land but also poses environmental risks, including dust emissions and groundwater contamination [16]. Fly ash possesses a large specific surface area and abundant reactive silica-alumina components, enabling effective physical adsorption and chemical immobilization of petroleum contaminants [17]. The synergistic use of lime and fly ash thus achieves the dual goal of “treating waste with waste” and efficient soil remediation, offering both economic and environmental benefits.

Regarding the specific proportions of lime and fly ash, previous studies [18,19] have shown that for the treated oil-contaminated soil (TOCS), a formulation of 10% lime and 20% fly ash yields an unconfined compressive strength (UCS) that could meet the USEPA [20] requirement for S/S-treated waste intended for landfilling (minimum UCS of 350 kPa). Moreover, at this ratio, the adsorption efficiency for oil is highest, and the leachability of contaminants is minimized [21]. Therefore, this formulation demonstrates good applicability and superiority for treating oil-contaminated soil. Nevertheless, a critical knowledge gap remains. The long-term performance and environmental safety of the treated material under real engineering conditions depend largely on the stability of immobilized contaminants under varying water environments, such as rainfall infiltration, groundwater fluctuation, and humidity changes.

Therefore, this work aims to evaluate the residual oil concentration, water retention characteristics, and permeability of both untreated oil-contaminated soil (UOCS) and TOCS under different water environments. By integrating these migration data with established mechanical performance metrics, we provide a comprehensive assessment of the reusability of TOCS. The findings of this work will help address a key bottleneck in the field, thereby facilitating the confident and sustainable engineering application of oil-contaminated soil.

## 2. Materials and Methods

### 2.1. Test Materials

#### 2.1.1. Soil

**Figure 1:**
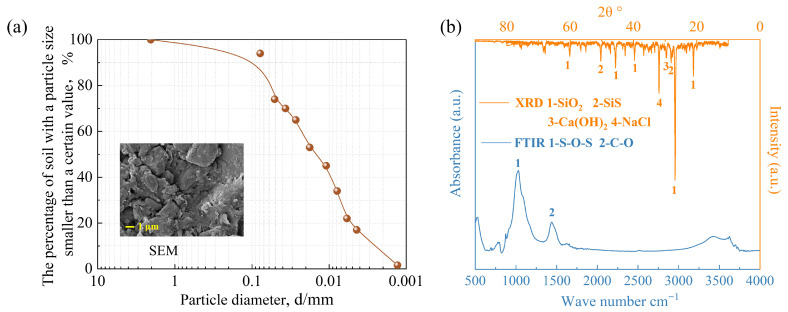
(**a**) Soil particle size and scanning electron microscopy image; (**b**) the XRD and FTIR patterns.

Soil was taken from the Binhai New Area of Tianjin, China, which is silty clay. Its physical and chemical properties are shown in Table 1, and its particle size and scanning electron microscopy image are shown in Figure 1a. The mineral composition was identified using X-ray diffraction (XRD) and Fourier transform infrared spectrum (FTIR) (Figure 1b). The XRD pattern confirmed that SiO_2_ is the dominant mineral, with small amounts of SiS and NaCl [22]. Concurrently, the infrared spectrum showed two characteristic bands for silicates (Si-O-Si stretching at 1030 cm^−1^) and carbonates (C-O stretching at 1442 cm^−1^) [23]. Therefore, the soil is primarily a silicate structure that incorporates inorganic carbonates.

#### 2.1.2. Oil

Oil was taken from the Dagang Oilfield in Tianjin, China. It appeared as a dark brown semi-fluid with a characteristic odor and belonged to the intermediate-base crude oil. Its basic physicochemical properties are summarized in Table 2.

#### 2.1.3. Solidified Materials

In this work, lime and fly ash were used as the solidification materials at addition amounts of 10% lime and 20% fly ash, which represent the optimal ratio for mechanical performance [18,19]. Among them, the lime was taken from Xinyang Calcium Industry Co., Ltd., Nanyang, China, which is quicklime. The effective calcium and magnesium content of it is 80%. Fly ash was taken from Tianjin Yangliuqing Power Plant (Tianjin, China). Its structure is a porous honeycomb, mainly composed of coal ash and slag (the mass ratio of coal ash to slag is 7:3). The average specific surface area is 0.812 m^2^/g. The main components of the lime and fly ash are shown in Table 3.

### 2.2. Specimen Preparation

The oil-contaminated soil was artificially configured. Determining the oil concentration parameters is critical for establishing the experimental protocol. If the oil concentration is too low, the study may not be sufficiently representative. If it is too high, oil exudation or leakage may occur during specimen compaction. Based on the contamination levels typically found at oil-contaminated sites and on previous studies [24,25,26,27,28] in the literature, the oil concentration range for this work was set from 30 to 150 mg/g, with detailed levels specified. Artificially prepared oil-contaminated soils with oil concentrations of 30, 60, 90, 120, and 150 mg/g were selected for testing.

The detailed preparation process of petroleum-contaminated soil is as follows: Firstly, the distilled water was mixed evenly into the soil, which was air-dried and sieved with a 2 mm filter net. Then it was placed in a closed container for 24 h. Secondly, the oil was mixed evenly into the soil and sealed for 24 h, and then the oil-contaminated soil was placed in a closed container before use.

As to the TOCS, lime and fly ash were mixed into the oil-contaminated soil, stirred evenly, and sealed again for 24 h. The static compaction method was used to prepare soil specimens. The treated soil specimens were placed in constant temperature (20 °C) and humidity (90 °C) conditions for 28 d.

### 2.3. Test Method

To comprehensively evaluate the performance of the TOCS under realistic and varied environmental exposures, three distinct water conditions were systematically investigated. This multi-scenario approach was designed to move beyond traditional static testing and probe the soil’s hydro-mechanical stability and contaminant retention capabilities across a spectrum of moisture transport mechanisms. The rationale and engineering relevance of each condition are as follows:

Humidity injection: Simulates water vapor transport in the vadose zone driven by changes in atmospheric humidity. This is essential for evaluating the long-term stability of surface TOCS.

Water injection: Simulates abrupt water intrusion events, such as heavy rainfall or pipe leakage. This tests the erosion resistance of the solidified material and the risk of contaminant release under rapid saturation.

Permeation: Simulates long-term conditions in the saturated zone under steady groundwater seepage. This is a classic method for assessing the long-term contaminant migration risk when S/S-treated material is used as a barrier or subgrade material.

By integrating these three distinct tests, this work covered a wide range of typical water environments from unsaturated to saturated and from instantaneous to long-term.

#### 2.3.1. Humidity Injection Test

**Figure 2:**
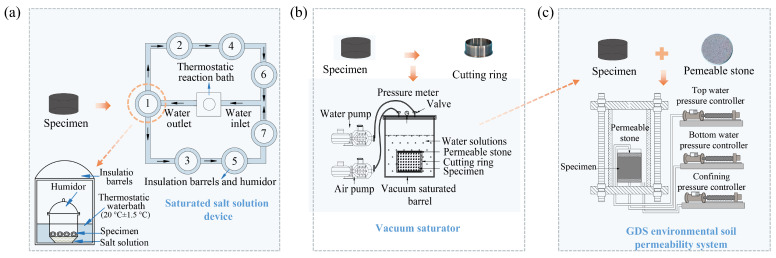
Test devices: (**a**) humidity injection test; (**b**) water injection test; (**c**) permeability test.

The migration situation of oil contaminants in UOCS/TOCS under varying humidity conditions was investigated using the saturated salt solution method [29]. A self-designed system, illustrated in Figure 2a, was employed for this test. The system consisted of a constant-temperature water bath and seven insulated barrels, which were connected in series via pipes to maintain a constant temperature inside the buckets. A humidor device was placed in each insulated bucket, containing the soil samples to be tested. A saturated salt solution was placed at the bottom of each device to create a stable suction environment, with the soil samples and the salt solution separated by a partition. A total of seven humidor devices were used in the experiment, each containing a different saturated salt solution to achieve varying relative humidity levels, thereby generating different capillary suctions within the soil samples. The types of saturated salt solutions, the corresponding humidity levels, and the associated capillary suction values are summarized in Table S1. The model and parameter details of the tested equipment are shown in Table S2.

During the testing process, the soil samples need to be placed in different moisture containers for the moisture absorption and desorption test to determine the migration changes in oil contaminants in the soil under the variation in soil matrix suction. At the same time, this experiment can also obtain the soil-water characteristic curve (SWCC) of UOCS/TOCS. The moisture absorption route was: (2.981 × 10^5^ kPa) → (1.14 × 10^5^ kPa) → (8.2 × 10^4^ kPa) → (3.8 × 10^4^ kPa) → (3.01 × 10^4^ kPa) → (1.42 × 10^4^ kPa) → (4.2 × 10^3^ kPa); the moisture desorption route was: (4.2 × 10^3^ kPa) → (1.42 × 10^4^ kPa) → (3.01 × 10^4^ kPa) → (3.8 × 10^4^ kPa) → (8.2 × 10^4^ kPa) → (1.14 × 10^5^ kPa) → (2.981 × 10^5^ kPa). (Note: The test parameter is matric suction).

#### 2.3.2. Water Injection Test

The water injection test was carried out by using a vacuum-saturated barrel, a vacuum pumping pump, and a water pump (Figure 2b). The permeable stone was placed with the sample on the upper and lower sides of the jackknife and placed into the vacuum-saturated barrel. To enhance the seal of the barrel, vaseline was applied between the barrel body and the cap. The vacuum pump was continuously pumped for 1 h (when the vacuum pressure gauge in the barrel showed 100 kPa), and then the water inlet valve was slowly adjusted to inject the water into the vacuum-saturated barrel. During the injection of water, the value of the pressure in the barrel should be kept constant by controlling the inlet valves and the pump. After the water sufficiently submerged the sample, the vacuum pump was turned off, the exhaust valve was opened, and air was allowed to enter the vacuum-saturated bucket for a period of 24 h, so that the sample was fully saturated with water. The model and parameter details of the tested equipment are shown in Table S2.

#### 2.3.3. Permeability Test

The permeability coefficient of contaminated soil was measured by the GDS environmental soil permeability system (Figure 2c). The model and parameter details of the tested equipment are shown in Table S2. The sample saturated by vacuum (as shown in Section 2.3.2) was used for the penetration test. The permeability system was equipped with three sets of digital pressure/volume controllers. One of them was used to apply the triaxial confining pressure, and the others were used to control the seepage head and the water head, respectively, and the permeability coefficient of the sample was automatically calculated. During the test, the osmotic pressure difference was set to 20, 40, 60, and 80 kPa, and the confining pressure was set to 200, 300, and 400 kPa.

#### 2.3.4. Determination of Oil in the Soil

The oil concentration in the soil samples after each test was determined by ultrasonic extraction followed by UV-Vis spectrophotometry [30]. The reliability and accuracy of this analytical method were rigorously validated for linearity, limit of detection, precision, and recovery. A detailed description of the method validation is provided in the Text S1 and Table S3.

**Figure 3:**
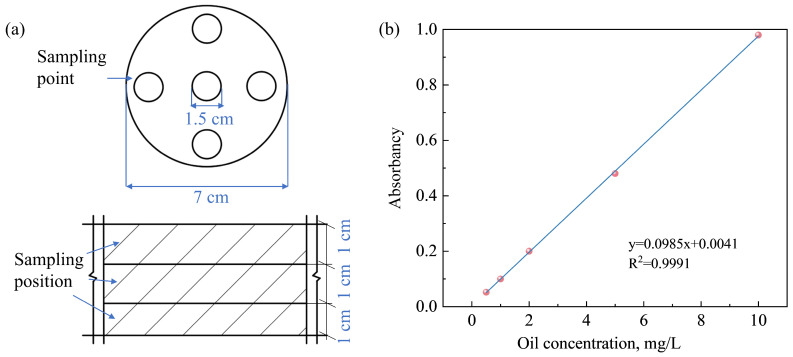
(**a**) Sampling position and sampling point; (**b**) standard curve.

For sample collection, a stratified sampling strategy was employed at 1 cm vertical intervals. To ensure each layer’s sample was representative, five different points within that layer (Figure 3a) were sampled. The collected composite samples were dried at 70 °C. Subsequently, a known mass of the dried soil was subjected to ultrasonic extraction with petroleum ether. After centrifugation, the supernatant was collected. The final oil concentration was then quantified at 225 nm against a standard curve (Figure 3b). To ensure the reproducibility of our findings, all experimental tests were conducted in triplicate, and the results are presented as mean values.

#### 2.3.5. Fitting of the Van Genuchten Model

The water retention characteristics of UOCS and TOCS were investigated by monitoring their water content changes over an equivalent matric suction range of approximately 4.2 × 10^3^–298.14 × 10^5^ MPa using the humidity injection test (saturated salt solution method). The resulting soil water retention behavior was quantitatively described by fitting the classical van Genuchten (VG) model to the experimental sorption data (Equation (1)) [31].

(1) $$\theta (\psi )=\theta_{r}+\frac{\theta_{s}-\theta_{r}}{{\left[1+{\left(\alpha \psi \right)}^{n}\right]}^{m}}$$

In the equation, *θ_r_* is the residual water content, *θ_s_* is the saturated water content, *α* is a parameter related to the inverse of the air-entry value, and *n* is a parameter reflecting the uniformity of the pore-size distribution (with the constraint *m* = 1 − 1/*n*).

The VG model was fitted to the experimental adsorption and desorption data for all soil samples to obtain the soil water retention curve parameters. The non-linear least-squares optimization method was used to determine the best-fit values for *θ_s_*, *θ_r_*, *α*, and *n*. The specific fitting process can be found in the supporting materials. The model showed excellent agreement with the experimental data across all samples, with coefficients of determination (R^2^) consistently above 0.98. The fitted parameters for representative samples are presented in Text S2, Tables S4 and S5.

## 3. Results and Discussion

### 3.1. Migration of Oil in the UOCS/TOCS Under the Different Water Environments

**Figure 4:**
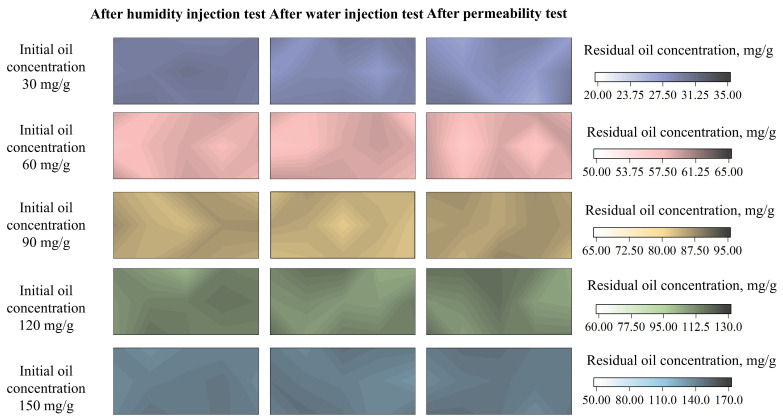
Spatial distribution of the final oil concentration in TOCS after different water environments.

To visualize the effect of the different water environments on UOCS/TOCS, the spatial distribution of oil concentration within the soil columns was mapped after the different water environments. The results for the TOCS are presented as contour maps in Figure 4; the corresponding distribution maps for the UOCS are provided in Figure S1. In these maps, it is important to note that the color at any given point represents the final, locally measured concentration of oil. The color gradient, therefore, illustrates the heterogeneous redistribution of oil throughout the soil column as a result of water flow.

**Figure 5:**
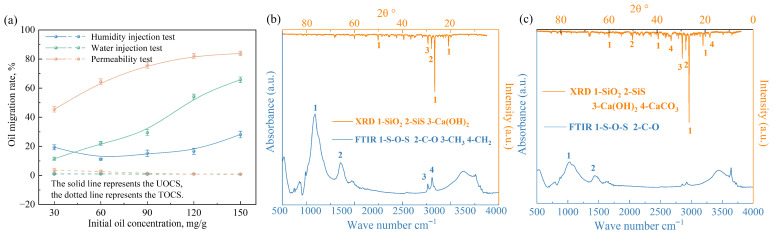
(**a**) The oil migration rate in the UOCS/TOCS under different water environments; (**b**,**c**) XRD and FTIR patterns of (**b**) UOCS and (**c**) TOCS.

As shown in Figure S1, different water environments have distinct effects on the migration and diffusion of oil within UOCS. The effect of humidity on the oil concentration change is minimal. The test method for humidity involves controlling internal soil matrix suction via saturated salt solutions, which is an internal force rather than an external one [32]. Therefore, this method results in low oil mobility, ranging only from 20.21% to 27.26% (Figure 5). Conversely, oil migration is far more pronounced under water injection and permeability conditions. The corresponding oil mobility was 23.21–63.67% for water injection and 42.03–84.01% for permeability. Notably, the residual oil concentration under the permeability test was lower than that under water injection. This can be attributed to the pre-saturation of the specimen with water before the permeability test, which enhances flushing. During this test, even with high initial concentrations of 90–150 mg/g, the final residual oil concentration consistently fell within a narrow range of 50–60 mg/g. This suggests that this value represents the soil’s maximum adsorption capacity for oil; any oil in excess of this capacity is easily desorbed and transported by the water flow.

The solidification treatment successfully limited the movement of oil within the soil. Across all water-based tests (humidity injection, water injection, and permeability) the final oil concentration in the TOCS remained very close to the initial amount, demonstrating a major drop in oil mobility compared to the UOCS (Figure 4). For instance, with a starting concentration of 150 mg/g, oil mobility in TOCS was reduced by 26.72% (humidity), 63.03% (water injection), and 83.47% (permeability), as detailed in Figure 5. The water injection test also provided clear visual confirmation: oil migrated to the surface in the UOCS, while the surface of the TOCS remained clean with no signs of seepage (Figure S2).

The underlying mechanisms for this effective containment are revealed through microstructural analyses. FTIR and XRD spectra (Figure 5b,c) showed that the oil contamination introduces characteristic peaks for CH_3_ and CH_2_ [33]. In the TOCS, the intensity of these peaks is significantly diminished, indicating that the solidifying agents effectively adsorb and encapsulate the oil contaminants. Concurrently, the FTIR for TOCS shows a marked decrease in the Si-O-Si peak. This is because the pozzolanic reaction during solidification converts a portion of the soil’s native organosilicon into C-S-H gel [34]. Further evidence from the XRD patterns confirms the formation of these new binding products, with a distinct enhancement of CaCO_3_ crystal diffraction peaks and the emergence of new Ca(OH)_2_ peaks in the TOCS. The Ca(OH)_2_, formed from the hydration of lime, is crucial as it provides the necessary alkaline environment (OH^−^) for the pozzolanic reactions to proceed [35]. Ultimately, the abundant formation of these cementitious products reinforces the soil’s structure, rendering it highly resistant to water and effectively controlling the migration of contaminants by physically locking them within the solidified matrix.

### 3.2. Water-Holding Capacity of the UOCS/TOCS Under the Humidity Injection

To systematically evaluate the effects of oil contamination and stabilization on soil water retention, the water content of UOCS and TOCS was monitored over a wide equivalent matric suction range of 4.2 to 298.14 MPa using the vapor equilibrium technique. The resulting data were used to analyze their water-holding capacity. The results reveal that UOCS exhibits a significantly poor water-holding capacity. Soil-water characteristic curves (SWCCs) fitted with the classical VG model show that at low matric suctions (<38 MPa), the water content of UOCS declines steeply (Figure S3). This indicates a low air-entry value and a reduced volume fraction of pores available for effective water adsorption and storage. The underlying mechanism is that the oil phase not only occupies pore space but also coats mineral surfaces, forming a hydrophobic film. This film is critical for water retention [36].

**Figure 6:**
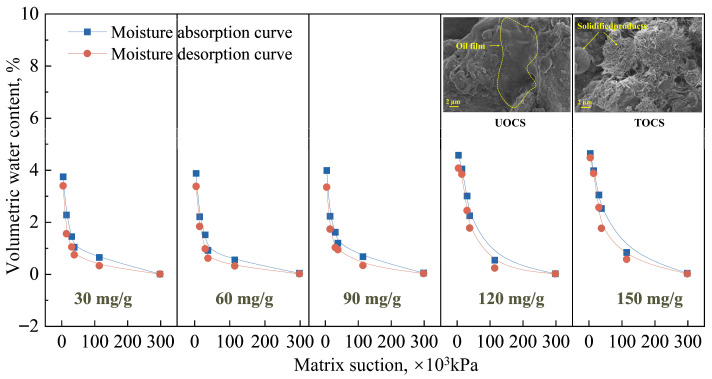
The SWCC of the TOCS.

Hysteresis was present in both UOCS and TOCS, but the effect was considerably more pronounced in UOCS (Figure S3 and Figure 6). With increasing initial oil concentration, the SWCC of UOCS shifted downward, and the area enclosed by the hysteresis loop decreased. This suggests that fewer pores participate in reversible wetting–drying cycles and that the divergence between the wetting and drying paths is diminished. This trend can be attributed to the oil phase occupying additional adsorption sites and imparting stronger hydrophobicity to the soil matrix, which restricts water entry into the pore network and limits its reconfiguration during cycling [37].

In contrast, lime–fly ash stabilization markedly improved the water-holding capacity of UOCS and attenuated the hysteresis in TOCS (Figure 6). This improvement stems from a dual synergistic mechanism. First, the solidified materials physically adsorb and chemically encapsulate the oil phase, reducing the system’s viscosity and surface water repellency, which in turn facilitates wetting. Second, pozzolanic reactions between lime and fly ash generate abundant needle- and rod-like cementitious products, such as C-S-H [38]. These products interweave within the soil pores to form a stable three-dimensional network that not only firmly immobilizes the oil but also refines and connects the pore structure by filling large voids, increasing the proportion of micropores, and creating new hydrophilic surfaces. Consequently, the VG curves of the stabilized soil shift upward across the entire suction range, and the residual water content increases. As a result, even at a high matric suction approaching 300 MPa, the treated soil maintains a higher bound water content and a more stable pore structure, rendering it less susceptible to adverse modification under environmental changes.

### 3.3. Permeability Characteristics of the UOCS/TOCS

**Figure 7:**
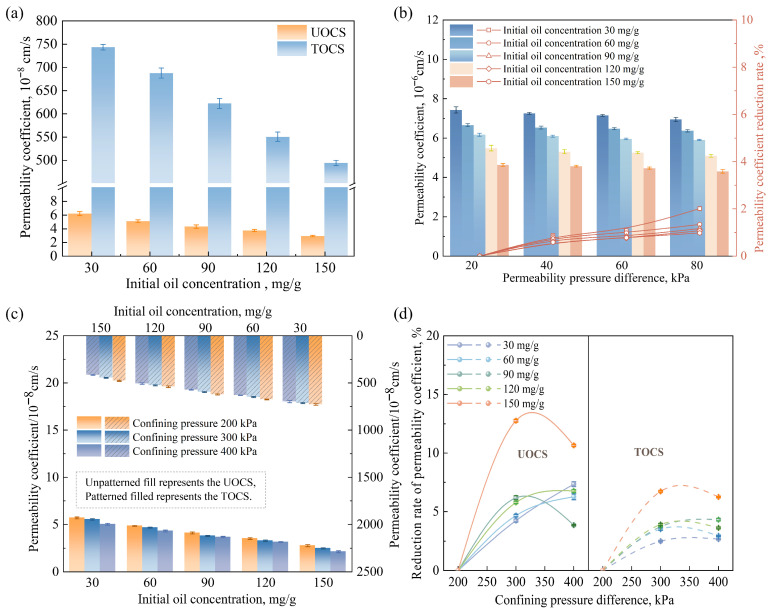
(**a**) Permeability coefficient of the TOCS at different initial oil concentrations; (**b**) permeability coefficient of the TOCS at different permeability pressure differences. The effect of permeability coefficient on the UOCS/TOCS at different pressure differences: (**c**) permeability coefficient; (**d**) permeability coefficient reduction rate.

**Figure 8:**
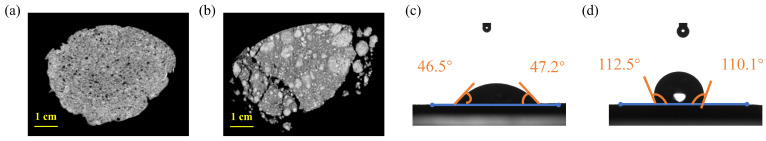
(**a**,**b**) CT images of (**a**) UOCS and (**b**) TOCS; (**c**,**d**) contact angle of (**c**) UOCS and (**d**) TOCS.

The permeability coefficients of UOCS and TOCS under varying initial oil concentrations are presented in Figure 7a. For UOCS, the permeability coefficient ranges from 2.24 × 10^−8^ to 5.81 × 10^−8^ cm/s. As the oil concentration increases, the permeability coefficient gradually decreases. This decline is primarily attributed to the physical blockage of soil pore throats by viscous oil, which increases the tortuosity of water flow paths and reduces the effective pore channels available for water molecule permeation [39]. Strikingly, the permeability coefficient of TOCS (4.28 × 10^−6^ to 7.39 × 10^−6^ cm/s) is approximately two orders of magnitude higher than that of UOCS. To further investigate the reason for this enhanced permeability in oil-contaminated soil after S/S treatment with lime and fly ash, CT scanning and contact angle measurements were conducted on both UOCS and TOCS (Figure 8). The results show that S/S treatment reduces the total porosity of the soil; moreover, the surface of TOCS (111.3°) is substantially more hydrophobic than that of UOCS (46.9°).

The reason for the increased permeability of TOCS might be due to the internal structural reorganization of the soil caused by lime and fly ash treatment. Specifically, the pozzolanic reaction promotes flocculation and cementation of fine soil particles, binding them into larger, structurally stable aggregates [40]. This process transforms the soil into a dual-porosity system: it eliminates numerous oil-clogged, ineffective micropores (hence the reduced total porosity), while simultaneously creating a new, well-connected network of inter-aggregate macropores. According to the Hagen–Poiseuille law, permeability is proportional to the fourth power of the pore radius [41]. Consequently, a few newly formed macropores can act as preferential flow paths, contributing far more to the overall permeability than the sum of countless micropores. Therefore, even though TOCS has lower total porosity and a more hydrophobic surface, water can still permeate through these efficient conductive channels, ultimately leading to a substantial increase in the measured permeability coefficient.

This enhanced structural integrity also explains the differential responses of the two soils under osmotic and confining pressures. As shown in Figure 7b and Figure S4, the permeability of both soils decreases with increasing osmotic pressure, likely due to soil compression [42]. However, at higher oil concentrations (90–150 mg/g), UOCS exhibits greater sensitivity to this effect (reduction rate of 2.47–4.48%), whereas TOCS shows lower sensitivity (reduction rate of 1.38–2.02%). This is because the cemented aggregate structure of TOCS possesses superior mechanical stability, enabling it to resist compression more effectively than the soft, oil-laden matrix of UOCS. Similarly, under increasing confining pressure (Figure 7c), the cemented skeleton of TOCS provides stronger resistance to mechanical compaction. This is evidenced by its lower reduction rate in permeability coefficient (2.54–6.27%), compared with the much higher reduction rate of UOCS (3.92–11.45%, Figure 7d). The robust inter-aggregate structure of TOCS maintains its overall integrity, whereas the poorly structured UOCS undergoes more pronounced pore collapse under stress.

## 4. Engineering Reusability and Environmental Safety Assessment

### 4.1. Synergistic Immobilization Mechanisms

**Figure 9:**
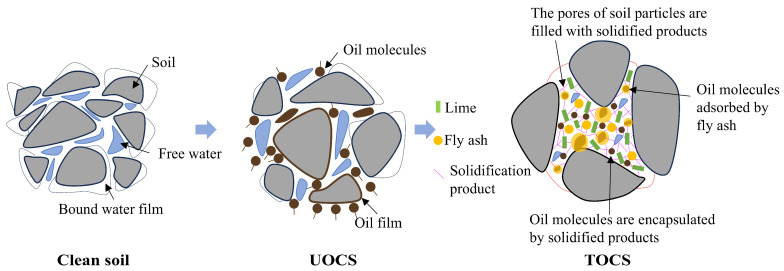
The mechanism of lime and fly ash solidified the oil-contaminated soil.

Lime–fly ash can effectively control the migration of oil pollutants in soil, which is the result of the combined effect of physical interception and adsorption fixation. During the treatment process, the hydration of lime and fly ash triggers the fly ash reaction, generating complex gels, mainly C-S-H and C-A-S-H gels. These gels not only fill the spaces but also form a microcrystalline network that covers soil particles, oil droplets, and other substances. This encapsulation not only forms a solid physical barrier but also effectively locks the pollutants within a dense mineral framework. In addition to simple physical closure, the huge surface area and active sites of these hydrated products provide another defense mechanism: they chemically fix the oil pollutants through adsorption, significantly reducing the fluidity of the oil phase (Figure 9).

### 4.2. Oil Migration

The test results show that the residual oil concentration of TOCS has significant stability, with the fluctuation range controlled within 2.3% (this finding is consistent with the stable range (0.3% to 4.9%) reported in similar rock property studies [43]). Interestingly, despite different water environments, this effective fixation phenomenon still exists. This indicates that the migration of oil in the TOCS and the overall water advection are independent of each other. Instead, the transport is likely controlled by the slow rate of diffusion through the pore solution. The cementing matrix forms a tortuous, disconnected pore structure, forcing the contaminants to follow an extremely tortuous path, thereby significantly slowing down their release [44].

Although this study did not model the complete advection–diffusion spectrum, the stability of the petroleum indicates that any potential fluxes follow the Fickian diffusion law [45]. Taking the cement-bound system as a reference, since lime–fly ash, like cement, undergoes pozzolanic reaction and the solidification products are basically the same, in cement-bound systems, effective diffusion coefficients for hydrocarbons typically drop to 10^−9^–10^−11^ cm^2^/s, nearly three to five orders of magnitude lower than in raw, untreated soils (10^−6^–10^−8^ cm^2^/s). These data can further confirm this point from another perspective: the lime–fly ash treatment method demonstrates effectiveness in preventing the leakage of substances in the environment.

### 4.3. Comparative Performance and Regulatory Compliance

The feasibility of repurposing contaminated soil hinges on whether it can function as a structural material without posing an environmental hazard. According to USEPA benchmarks, the results of TOCS could be used as structural materials (Table 4).

Mechanical integrity: The TOCS achieved UCS of 570.2–938.5 kPa. This is not merely a passing grade; it is nearly four times the USEPA threshold of 350 kPa, ensuring sufficient bearing capacity for heavy engineering loads.

Permeability control: Permeability coefficient ranged between 4.28 × 10^−6^ and 7.39 × 10^−6^ cm/s. Unlike cement, which can “over-solidify” and render soil brittle, or laterite, which offers minimal porosity reduction, the lime–fly ash blend strikes an optimal balance (Figure S5). It modifies the soil’s internal viscosity and hydrophobicity, yielding a material that meets the 10^−4^ to 10^−8^ cm/s requirement for engineered fills.

Leaching safety: Toxicity characteristic leaching procedure (TCLP) analysis for heavy metals (As, Ba, Cd, Cr, Pb, Se, and V) remained well below regulatory limits. The detailed steps of TCLP are provided in the Text S3.

The evidence presented here supports a shift from “treat-and-dump” to “stabilize-and-reuse.” By meeting the structural demands of UCS, the environmental safeguards of TCLP and the permeability mandated by the USEPA, lime–fly ash-solidified soil proves to be a viable candidate for roadbed and embankment construction. This approach effectively transforms a hazardous liability into a geotechnical asset, offering a sustainable pathway for waste valorization in infrastructure projects.

## 5. Conclusions

This work aims to analyze the effect of different water environments (water injection, humidity injection, and permeability) on the oil-contaminated soil co-solidified by lime and fly ash. With the help of a self-built water injection test device, a saturated salt solution device, and a GDS environmental soil permeability system, the migration and diffusion law of oil in the UOCS/TOCS under different water environments, as well as the water-holding capacity and the permeability characteristics of the soil, were analyzed. The main conclusions are drawn as follows:

(1) The co-solidification with lime and fly ash effectively controlled the migration and diffusion of oil, mitigating the variability caused by changes in dry density and oil concentration. Across the tested aqueous environments, the maximum oil mobility in TOCS ranged from 0.44% to 4.90%, which was substantially lower than the 12.54% to 85.54% range observed in UOCS.

(2) The lime and fly ash treatment was demonstrated to enhance the water-holding capacity of the oil-contaminated soil. This treatment also reduced the dependency of the water-holding capacity on soil density and diminished the hysteresis effect that was prominent in UOCS.

(3) The solidification process was found to reduce the water repellency of the oil-contaminated soil and increase effective permeation pathways. The permeability coefficient of the solidified soil ranged from 4.28 × 10^−6^ to 7.39 × 10^−6^ cm/s, a range that satisfies the criterion for stabilized/solidified materials (10^−4^–10^−8^ cm/s) as recommended in established resource guides.

(4) Lime and fly ash-co-solidified soil showed significant potential for engineering reuse as a fill material in applications such as road subgrades or embankments.

## Figures and Tables

**Table 1:** Physical and chemical indexes of soil.

Air Dry Moisture Content %	Consistency Index			Compaction Characteristics	
	Liquid Limit %	Plastic Limit %	Plasticity Index	Optimal Moisture Content, %	Maximum Dry Density, g/cm^3^
2.4	30.6	18.8	11.8	1.87	14.41

**Table 2:** The basic physicochemical properties of oil.

Category	Standard Density, kg/m^3^	State	The Content of Each Component, %			
			Saturated Hydrocarbon	Aromatic Hydrocarbon	Nonhydrocarbon	Asphaltene
Value	858	Semifluid	52.21	29.18	8.00	16.62

**Table 3:** The components of lime and fly ash.

Component	SiO_2_	Al_2_O_3_	Fe_2_O_3_	CaO	MgO	TiO_2_	P_2_O_5_	SO_3_
Lime	1.08	-	-	95.32	0.68	-	-	0.01
Fly ash	52.58	30.32	8.73	2.33	0.73	1.52	0.84	-

**Table 4:** Comparison results of TOCS parameters with USEPA.

	TCLP ppm							UCS kPa	Permeability Coefficient, cm/s
	As	Ba	Cd	Cr	Pb	Se	V		
TOCS	<0.01	0.247	<0.01	<0.01	<0.01	<0.02	<0.01	570.2–938.5	4.28 × 10^−6^–7.39 × 10^−6^
USEPA requirement	5	100	1	5	5	1	25	350	10^−4^–10^−8^

## Data Availability

The original contributions presented in this study are included in the article. Further inquiries can be directed to the corresponding author.

## Supplementary Materials

The following supporting information can be downloaded at: https://www.mdpi.com/article/10.3390/toxics14050357/s1, Text S1: Ultrasound–ultraviolet method; Text S2: van Genuchten model fitting and parameters; Text S3: Toxicity Characteristic Leaching Procedure test [20,46,47]; Table S1: Saturated salt solutions, relative humidity, and corresponding matric suction in each humidor; Table S2: The model and parameter details of the tested equipment; Table S3: Spike and recovery results for oil-contaminated soil samples (n = 3); Table S4: Fitted VG model parameters for UOCS samples at various oil concentrations; Table S5: Fitted VG model parameters for TOCS samples at various oil concentrations; Figure S1: Spatial distribution of the final oil concentration in TOCS after different water environments; Figure S2: Water injection test diagram; Figure S3: Hysteresis phenomenon of the TOCS; Figure S4: Permeability coefficient of the UOCS at different initial oil concentrations; Figure S5: Permeability coefficient of the clean soil, UOCS, and TOCS solidified with different solidification materials [48,49,50,51].

## Abbreviations

The following abbreviations are used in this manuscript:

C-A-H	Calcium aluminate hydrate
C-S-H	Calcium silicate hydrate
FTIR	Fourier transform infrared spectrum
S/S	Solidification/stabilization
TCLP	Toxicity characteristic leaching procedure
TOCS	Treated oil-contaminated soil
UCS	Unconfined compressive strength
UOCS	Untreated oil-contaminated soil
VG	Van Genuchten
XRD	X-ray diffraction

## References

[1] Al-Mebayedh H., Niu A., Lin C. (2023). Strategies for cost-effective remediation of widespread oil-contaminated soils in Kuwait, an environmental legacy of the first Gulf War. J. Environ. Manag..

[2] Dike C.C., Khudur L.S., Hakeem I.G., Rani A., Shahsavari E., Surapaneni A., Shah K.L., Ball A.S. (2022). Biosolids-derived biochar enhances the bioremediation of diesel-contaminated soil. J. Environ. Chem. Eng..

[3] Lv Y., Bao J., Zhu L. (2022). A comprehensive review of recent and perspective technologies and challenges for the remediation of oil-contaminated sites. Energy Rep..

[4] Ostovar M., Ghiassi R., Mehdizadeh M.J., Shariatmadari N. (2021). Effects of crude oil on geotechnical specification of sandy soils. Soil Sediment Contam..

[5] Wang Y., Chen Y., Chen Y., Chen D., Li Y., Qin J. (2024). Microemulsion phase behavior based on biodiesel and its performance in treating oil-contaminated soil. J. Environ. Chem. Eng..

[6] Park J. (2021). Evaluation of changes in the permeability characteristics of a geotextile-polynorbornene liner for the prevention of pollutant diffusion in oil-contaminated soils. Sustainability.

[7] Fang X., Zhang M., Zheng P.F., Wang H.M., Wang K.F., Lv J., Shi F.C. (2024). Biochar-bacteria-plant combined potential for remediation of oil-contaminated soil. Front. Microbiol..

[8] Zhao Y.X., Sun Y.H., Sun H.H., Zuo F., Kuang S.P., Zhang S., Wang F.Y. (2024). Surfactant-based chemical washing to remediate oil-contaminated soil: The state of knowledge. Toxics.

[9] An S., Woo H., Kim S.H., Yun S.-T., Chung J., Lee S. (2023). Complex behavior of petroleum hydrocarbons in vadose zone: A holistic analysis using unsaturated soil columns. Chemosphere.

[10] Chen H., Hao Y., Zhang S., Pan J.R., Lang M., Guo X. (2023). Vertical migration and variation of crude oil in soil around typical oilfields under natural leaching. Int. J. Environ. Sci. Technol..

[11] Luo Z., Tang C., Hao Y., Wang Z., Yang G., Wang Y., Mu Y. (2022). Solidification/stabilization of heavy metals and its efficiency in lead-zinc tailings using different chemical agents. Environ. Technol..

[12] Ma F., Wu B., Zhang Q., Cui D., Liu Q., Peng C., Li F., Gu Q. (2018). An innovative method for the solidification/stabilization of PAHs-contaminated soil using sulfonated oil. J. Hazard. Mater..

[13] Giordano F.G., Etcheverry J.M., Brunin M., Boon N., Rodriguez-Navarro C., De Belie N. (2025). Evaluating the self-healing capacity of lime and lime-based Mortars: Effects of age, curing conditions, formulation and testing methods. J. Build. Eng..

[14] Silva B.A., Guerreiro E., Duarte A.P.C. (2025). Comparative study of lime-based mortars for conservation and restoration interventions. Constr. Build. Mater..

[15] Shirin S., Jamal A., Emmanouil C., Singh V.P., Yadav A.K. (2023). Assessment and characterization of waste material used as backfilling in an abandoned mine. Int. J. Coal Prep. Util..

[16] Suwan T., Fan M.Z., Wong H.S., Jitsangiam P., Hansapinyo C., Chindaprasirt P. (2025). Influence of ammonia-contaminated fly ash from selective catalytic reduction process on the properties of Portland-fly ash blended cement and geopolymer composites. Case Stud. Constr. Mater..

[17] Krishnan A.K., Wong Y.C., Zhang Z.P., Arulrajah A. (2024). A transition towards circular economy with the utilisation of recycled fly ash and waste materials in clay, concrete and fly ash bricks: A review. J. Build. Eng..

[18] Ezeokpube G.C., Ahaneku I.E., Alaneme G.U., Attah I.C., Etim R.K., Olaiya B.C., Udousoro I.M. (2022). Assessment of mechanical properties of soil-lime-crude oil-contaminated soil blend using regression model for sustainable pavement foundation construction. Adv. Mater. Sci. Eng..

[19] Li M., Ma C., Sun Z., Yao X. (2022). Mechanical properties distribution of lime-fly ash solidified oil contaminated soil in a coastal environment. Eur. J. Environ. Civ. Eng..

[20] USEPA (1999). Solidification/Stabilization Resource Guide.

[21] Li Y., Zeng X., Zhou J., Liu H., Gu Y., Pan Z., Zeng Y., Zeng Y. (2020). Incorporation of disposed oil-contaminated soil in cement-based materials. Resour. Conserv. Recycl..

[22] Na S.Y., Jeong H., Kim I., Hong S.M., Shim J., Yoon I.H., Cho K.H. (2024). Distribution coefficient prediction using multimodal machine learning based on soil adsorption factors, XRF, and XRD spectrum data. J. Hazard. Mater..

[23] Rathikannu S., Gautam S., Joshi S.K., Katharine P., Mithra K.E., Banusaranya P., Amudhavarshini V.M., Gayatri R., Ho C.H. (2025). FTIR based assessment of microplastic contamination in soil water and insect ecosystems reveals environmental and ecological risks. Sci. Rep..

[24] Balkaya M. (2019). A parametric study on numerical simulation of crude oil contaminated site capping. Desalin. Water Treat..

[25] Mustafa Y.M.H., Al-Amoudi O.S.B., Ahmad S., Maslehuddin M., Al-Malack M.H. (2021). Utilization of Portland cement with limestone powder and cement kiln dust for stabilization/solidification of oil-contaminated marl soil. Environ. Sci. Pollut. Res..

[26] Kogbara R.B., Al-Tabbaa A., Yi Y., Stegemann J.A. (2013). Cement-fly ash stabilisation/solidification of contaminated soil: Performance properties and initiation of operating envelopes. Appl. Geochem..

[27] Lei X., Yang M., Qin B., Jiang J.L., Shen Y.T., Qiao F.L. (2025). Performance and mechanism of enhanced washing of crude oil-contaminated soil using sophorolipid and its esterified derivative. J. Environ. Chem. Eng..

[28] Jiang S., Wang L., Wang S., Liang J.L., Lu G., Li L., Zhang Y., Wang Q.H., Zhang L.Q. (2025). Kinetics and solid effect investigations during oil droplet desorption from oil-contaminated soil using the chemical cleaning method. Molecules.

[29] Zheng Y.P., Zhang X.D., Zhang J.C., Liu H.C. (2024). A polyimide-coated fiber bragg grating sensor for multidepth soil humidity measurement. IEEE Sens. J..

[30] Fiastru-Irimescu M., Ene D., Margina D. (2024). Characterization of some plant extracts by ultrasound-assisted extraction in sunflower oil using thin layer chromatography and spectrophotometry UV-vis. Stud. Univ. Babes-Bolyai Chem..

[31] Ghorbani A., Babaeian E., Sadeghi M., Durner W., Jones S.B., van Genuchten M.T. (2025). An improved van Genuchten soil water characteristic model to account for surface adsorptive forces. J. Hydrol..

[32] Shoormasti N.H., Tabatabaei-Nezhad S.A. (2022). A novel mechanistic anion exclusion model to investigate partially water-saturated transport in soils and shales: A case study of nitrate solution flow. Eur. J. Soil Sci..

[33] Aakula M., Kedare M.M., Patra S., Gangar T., Singha S., Meher S., Sahoo L., Rao S., Radhakrishnanand P. (2026). Restoration of crude oil-contaminated soil using microbes with degradative and plant-growth-promoting abilities. J. Microbiol. Methods.

[34] Arabani M., Ranjbar P.Z., Haghsheno H. (2025). Controlling compressibility in oil-contaminated soils using alkali-activated slag: A sustainable approach. Int. J. Environ. Sci. Technol..

[35] Petrillo A., Fraternali F., Acampora A., Di Chiara G., Colangelo F., Farina I. (2025). Innovative solidification and stabilization techniques using industrial by-products for soil remediation. Appl. Sci..

[36] Nasiri H., Khayat N., Nazarpour A. (2024). Utilization of the oil-contaminated soil as a sustainable resource in rural road construction and rehabilitation in oil-producing countries. J. Clean. Prod..

[37] He L., Wang Z., Gu W. (2021). Evolution of freeze-thaw properties of cement-lime solidified contaminated soil. Environ. Technol. Innov..

[38] Wang T., Medepalli S., Zheng Y.Q., Krishnan S., Li N., Ishida T., Zhang Z.H., Bishnoi S., Zhang K. (2024). An efficient method for determining the pozzolanic reaction degrees of low-calcium supplementary cementitious materials in blended cement pastes. Cem. Concr. Compos..

[39] Pei W.S., Ye Z.L., Zhang M.Y., Lu J.G., Zhou J.Z., Liu W.B. (2025). Experimental and numerical investigations on spilled oil migration and contamination characteristics in freezing soils. Geoderma.

[40] Wang T., Medepalli S., Zheng Y.Q., Zhang W., Ishida T., Bishnoi S., Hou D.S., Shi Z.G. (2024). Retardation effect of the pozzolanic reaction of low-calcium supplementary cementitious materials on clinker hydration at later age: Effects of pore solution, foreign ions, and pH. Cem. Concr. Res..

[41] Zimparov V.D., Petkov V.M., Hristov H.N. (2021). Optimal spacings for channels with hagen-poiseuille fluid-flow and mass transfer-the role of the bejan number. Therm. Sci..

[42] Wang S.Y., Ling F.L., Hu Q.X., Qu T.M., Shang J.L. (2025). Effect of water pressure on time-dependent permeability characteristics of sand conditioned with foam and bentonite slurry. Can. Geotech. J..

[43] Li M., Yu H., Zheng D., Klemes J.J., Wang J. (2021). Effects of salt and solidification treatment on the oil-contaminated soil: A case study in the coastal region of Tianjin, China. J. Clean. Prod..

[44] Long H., Liao Y., Cui C.H., Liu M.J., Liu Z.W., Li L., Hu W.Z., Yan D.H. (2022). Assessment of popular techniques for co-processing municipal solid waste in Chinese cement kilns. Front. Environ. Sci. Eng..

[45] Cohen M., Berkowitz B. (2026). Exploring asymptotically-Fickian chemical transport in porous media. J. Contam. Hydrol..

[46] USEPA (1990). EPA Method 1311.

[47] USEPA (2007). Method 8015C: Nonhalogenated Organics by Gas Chromatography.

[48] Abdulhamid S.N., Hasan A.M., Aziz S.Q. (2021). Solidification/stabilization of contaminated soil in a south station of the khurmala oil field in Kurdistan Region. Iraq. Appl. Sci..

[49] Abdelhalim R.A., Selamat M.R., Ramli H. (2022). Evaluation of strength properties of oil-contaminated sands upon stabilisation with laterite soil. Int. J. Pavement Eng..

[50] Ahmad S., Al-Amoudi O.S.B., Mustafa Y.M.H., Maslehuddin M., Al-Malack M.H. (2020). Stabilization and solidification of oil-contaminated sandy soil using portland cement and supplementary cementitious materials. J. Mater. Civil Eng..

[51] Ahmad S., Ba-Naimoon M.S.M., Bahraq A.A., Al-Amoudi O.S.B., Maslehuddin M., Al-Malack M.H. (2022). Stabilization/solidification of petroleum oil-contaminated soil using different sabilizers to deliver a pavement subbase material. Arab. J. Sci. Eng..

