# Supplementary material for: Lime and Fly Ash Co-Solidification Treatment of Oil-Contaminated Soil: Characteristics in Different Water Environments and Evaluation of Engineering Reuse
Source: Toxics. 2026 Apr 23;14(5):357. doi: 10.3390/toxics14050357 (PMC13211010; doi:10.3390/toxics14050357)
Supplement: Supplementary file 1 [file toxics-14-00357-s001.zip › toxics-4236426-supplementary.pdf]

Supplemental Information

Lime and fly ash co-solidification treatment of oil-contaminated soil: Characteristics in different water environments and evaluation of engineering reuse

Hemiao Yu [1,†], Pei Gao [2,3,†], Hui Li [1] , Min Li [1,*]

[1] College of Civil Engineering and Transportation, Hebei University of Technology, Tianjin 300401, China.
[2] Key Laboratory for Green Chemical Technology of Ministry of Education, School of Chemical Engineering and Technology, Tianjin University, Tianjin 300072, China.
[3] Ningbo Key Laboratory of Green Petrochemical Carbon Emission Reduction Technology and Equipment, Zhejiang Institute of Tianjin University, Ningbo 315201, China.
* Correspondence: limin0409@hebut.edu.cn
† These authors contributed equally to this work.

Text S1. Ultrasound–ultraviolet method

S1.1. Validation of the ultrasound–ultraviolet analytical method

To ensure the accuracy and reliability of the quantitative data, the ultrasound–ultraviolet analytical method was comprehensively validated. The validation covered the assessment of linearity, limit of detection (LOD), limit of quantification (LOQ), precision, and accuracy over the concentration ranges relevant to this study.

S1.2. Linearity and calibration curve

First, an oil stock solution of 100 mg/L was prepared by dissolving oil in petroleum ether. A series of dilutions with petroleum ether were then performed to obtain standard working solutions at concentrations of 0.5, 1.0, 2.0, 5.0, and 10.0 mg/L. The absorbance of each standard solution was measured in triplicate at the maximum absorption wavelength (λ_{max}=225 nm). The calibration curve showed excellent linearity over this range, with the linear regression equation y=0.0985x+0.0041 and a correlation coefficient (R^2) of 0.9991. The corresponding calibration graph is presented in Figure S1. This concentration range (0.5-10.0 mg/L) defines the effective linear working range of the instrument.

S1.3. Limit of detection (LOD) and limit of quantification (LOQ)

The LOD and LOQ were determined based on the standard deviation of 11 blank sample measurements (petroleum ether extracts obtained from clean, analyte-free soil).

Using Eqs. (1) and (2), the calculated LOD was 0.06 mg/L and the LOQ was 0.18 mg/L.

$$LOD = 3.3 \times (\sigma / S) \quad \text{(S1)}$$

$$LOQ = 10 \times (\sigma / S) \quad \text{(S2)}$$

In the equation, σ is the standard deviation of the blanks and S is the slope of the calibration curve.

S1.4. Precision, accuracy, and matrix effects

Blank soil samples were spiked with three representative oil concentration levels covering the whole study range: low (30 mg/g), medium (90 mg/g), and high (150 mg/g). Three replicate samples were prepared for each concentration level. The samples were first extracted using the standard ultrasonic extraction procedure. For high-concentration samples, a quantitative dilution was performed before measurement. The final diluted solutions were then analyzed. Recoveries and relative standard deviations (RSDs) were calculated, taking the dilution factor into account. As shown in Table S1, the mean recoveries ranged from 97.5% to 99.3%, and all RSD values were below 5.0%. These results confirmed that the overall analytical method was accurate, precise, and free of significant matrix effects.

Text S2.van Genuchten model fitting and parameters

The van Genuchten (V-G) model was fitted to the experimental adsorption (wetting) and desorption (drying) data for all soil samples to obtain the soil–water retention curve parameters. The non-linear least-squares optimization method was used to determine the best-fit values for saturated water content (θ_s), residual water content (θ_r), the air-entry parameter (α), and the pore-size distribution index (n). The model showed excellent agreement with the experimental data across all samples, with coefficients of determination (R^2) consistently above 0.98.

The parameters in Table S4 and Table S5 quantitatively support the conclusions drawn in the main text. For the UOCS samples, an increase in oil concentration leads to a systematic decrease in both θ_s and θ_r, an increase in α (indicating a lower air-entry value),

and a slight decrease in n, reflecting a less uniform pore structure. Conversely, the stabilization treatment (TOCS) consistently results in higher θ_s and substantially higher θ_r values compared to their UOCS counterparts at the same oil concentration. Furthermore, the α values for TOCS are significantly lower and the n values are higher, confirming a refined pore structure with improved water retention at high suction.

Text S3. Toxicity Characteristic Leaching Procedure test

Some samples were considered for the TCLP test. The TCLP test was carried out according to the USEPA 1311 method to detect heavy metals that might be in the TOCS (USEPA 1990 [S1]). These samples were crushed to obtain the size required by the USEPA 1311 method (<9.5 mm). After that, about 5 grams of each mix was combined with 96.5 mL of distilled water, then agitated using a magnetic agitator. After agitation, the pH of the mixes was measured using a pH meter with 0.05 accuracy. The pH for all samples was found to be more than 5. Therefore, 3.5 mL of 1N HCl was added to the mixes, they were heated to 50 °C, and the pH was measured again. The pH was still found to be higher than 5; hence, the extraction fluid was prepared by diluting 5.7 mL of glacial CH_3CH_2OOH with distilled water to a volume of 1 liter to reduce the pH of the sample (acidification). Ten grams of each soil sample was added to 200 mL of the prepared extraction fluid inside a fiberglass container. After preparing the samples, they were placed in a mechanical rotary agitator that runs at about 30 rpm for 18 h.

Thereafter, the samples were filtered using a vacuum filter, and the liquid extracts were collected. These samples were analyzed for the concentration of heavy metals using an inductively coupled plasma photometer (ICP).

The TPH (total petroleum hydrocarbons) test was carried out on the same samples that were considered for the TCLP test. The preparation of the samples was carried out according to USEPA method 3545 (USEPA1998 [S2]), where 5 g of each sample was collected and mixed with 25 mL of a hexane:dichloromethane solvent. The mixture was left for 24 h to allow for the full reaction between the solvent and hydrocarbons in the soil samples. Then, the resulting solution was collected, and another 10 mL of the solvent was added to the remaining sediments inside the flask, left for another 24 h, and thereafter added to the previous solution. The samples were left for about 8 h to allow the sediments to settle, and then 1.5 mL was collected from each sample and placed inside a small test tube. The samples were analyzed according to USEPA method 8015C using a gas chromatograph mass spectrometer (GCMS) device, and the results were recorded (USEPA 2007 [S3]).

Table S1. Saturated salt solutions, relative humidity, and corresponding matric suction in each humidor.

Humidor	Solution	Relative humidity (RH), %	Suction, ×10³kPa
1	K_2SO_4	97	4.2
2	$ZnSO_4$	90	14.2
3	$(NH4)SO_4$	80	30.1
4	NaCl	75	38.0
5	$Mg(NO_3)_2$	55	82.0
6	K_2CO_3	43	114.0

Table S2. The model and parameter details of the tested equipment.

Test	Device name	Quantity	Model	Manufacturer, city	Country of origin
**Humidity injection test**	Constant Temperature Water Bath	1	HWS-28	Yiheng (Shanghai) Instrument Co., Ltd.	Shanghai, China
	Insulated Holding Tank	7	Custom (20 L, 304 stainless steel, double-wall)	Custom fabrication	Guangzhou, China
	Humidifier	7	YZ-MG8 (industrial ultrasonic)	Yadu (Beijing)	Beijing, China
**Water injection test**	Water Pump	2	CDLF2-22	CNP (Nanfang Pump)	Hangzhou, China
	Vacuum Saturation Chamber	1	Custom (304 stainless steel, φ500×600 mm)	Custom fabrication	Nanjing, China
**Permeability test**	GDS Testing System	1	GDSRTS (3-channel pressure/volume controller system)	GDS Instruments Ltd.	Hook, Hampshire, United Kingdom

Table S3. Spike-and-recovery results for oil-contaminated soil samples (n=3).

Spiking level	Spiked concentration (mg/g)	Dilution factor	Recovery (%)	RSD (%)
Low	30	100	97.5	4.6
Medium	90	500	98.8	3.1
High	150	500	99.3	2.7

Table S4. Fitted VG model parameters for UOCS samples at various oil concentrations.

Oil concentration, mg/g	Path	θ_s, cm^3/cm^3	θ_r, cm^3/cm^3	α, MPa^{-1}	n	R^2
30	Adsorption	0.392	0.046	0.022	1.39	0.992
	Desorption	0.395	0.058	0.015	1.36	0.995
60	Adsorption	0.385	0.041	0.026	1.35	0.991
	Desorption	0.388	0.052	0.019	1.31	0.994
90	Adsorption	0.373	0.035	0.030	1.32	0.990
	Desorption	0.377	0.044	0.022	1.29	0.993
120	Adsorption	0.362	0.030	0.033	1.30	0.988
	Desorption	0.366	0.038	0.024	1.27	0.991
150	Adsorption	0.351	0.026	0.035	1.28	0.989
	Desorption	0.355	0.034	0.025	1.25	0.992

Table S5. Fitted VG model parameters for TOCS samples at various oil concentrations.

Oil concentration, mg/g	Path	θ_s, cm^3/cm^3	θ_r, cm^3/cm^3	α, MPa^{-1}	n	R^2
30	Adsorption	0.421	0.108	0.009	1.51	0.997
	Desorption	0.423	0.115	0.006	1.49	0.998
60	Adsorption	0.412	0.095	0.011	1.48	0.996
	Desorption	0.415	0.103	0.008	1.45	0.997
90	Adsorption	0.405	0.091	0.013	1.45	0.996
	Desorption	0.408	0.098	0.009	1.42	0.995
120	Adsorption	0.401	0.089	0.014	1.43	0.994
	Desorption	0.404	0.096	0.010	1.41	0.996
150	Adsorption	0.398	0.088	0.015	1.42	0.995
	Desorption	0.401	0.094	0.011	1.40	0.996

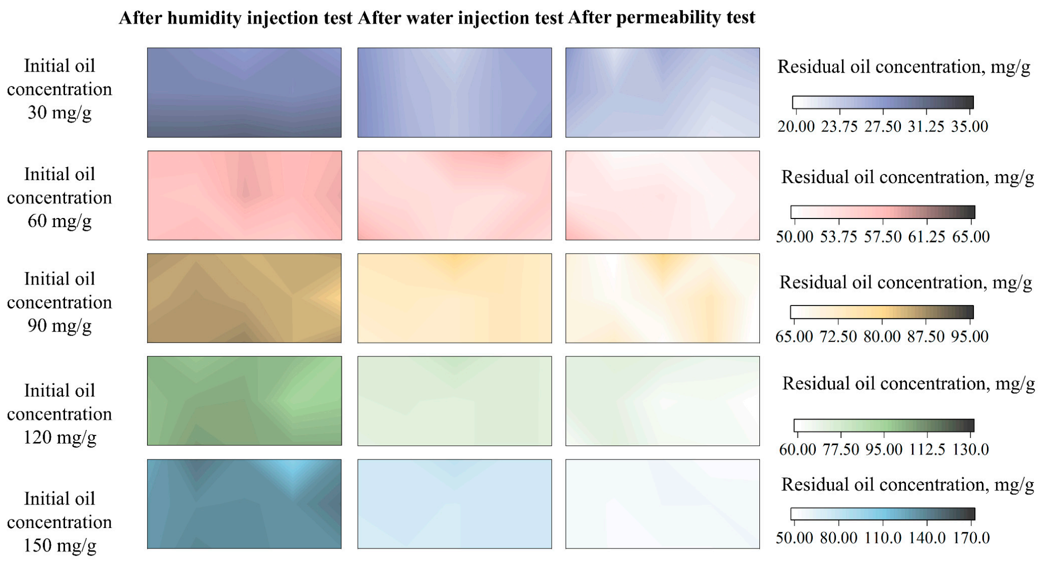

Figure S1. Spatial distribution of the final oil concentration in TOCS in different water environments.

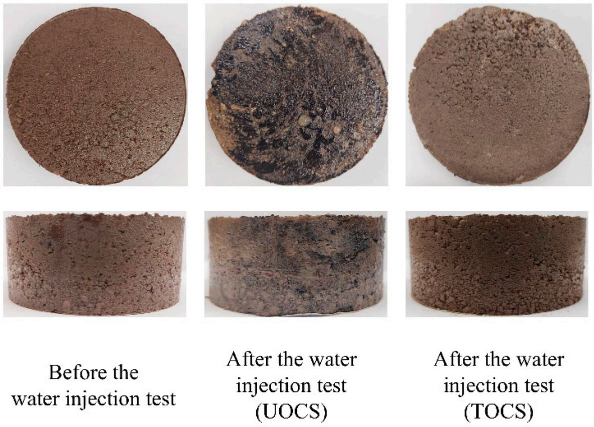

Figure S2. Water injection test diagram.

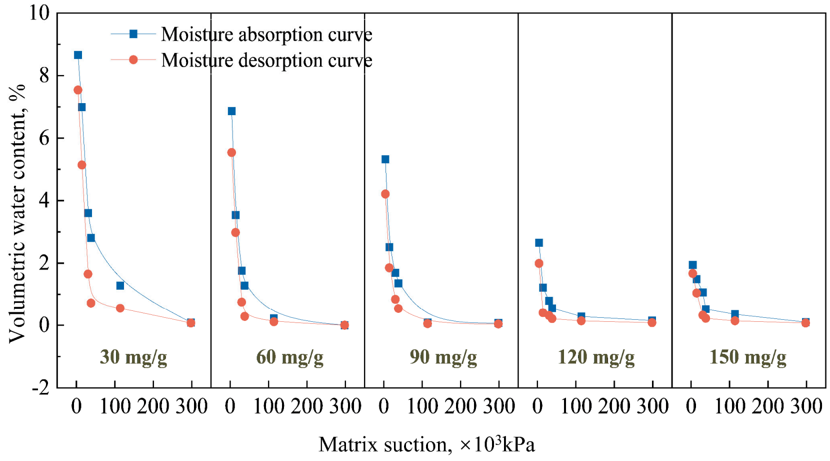

Figure S3. Hysteresis phenomenon of the TOCS.

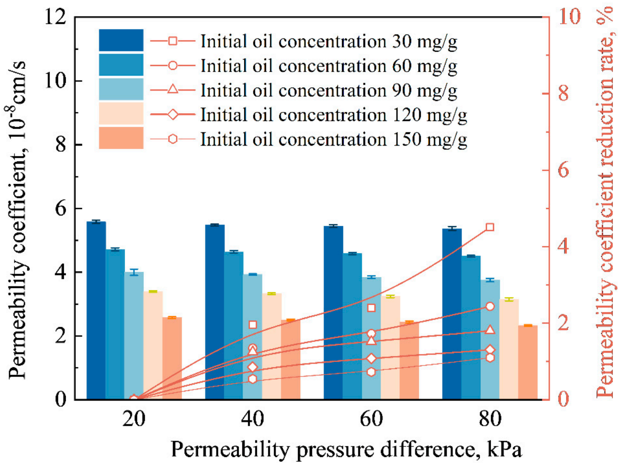

Figure S4. Permeability coefficient of the UOCS at different initial oil concentrations.

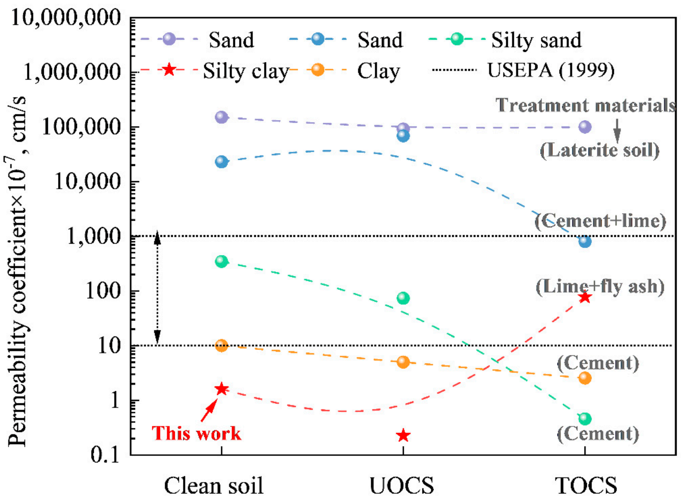

Figure S5. Permeability coefficient of the clean soil, UOCS, and TOCS solidified with different solidification materials [S4-S7].

References

[S1] USEPA. 1990. EPA method 1311. Washington, DC: USEPA.

[S2] USEPA. 1999. Solidification/stabilization resource guide. EPA/542-B-99-002. Washington, DC: USEPA.

[S3] USEPA. 2007. Method 8015C: Nonhalogenated organics by gas chromatography. Washington, DC: USEPA

[S4] Abdulhamid, S.N.; Hasan, A.M.; Aziz, S.Q. Solidification/stabilization of contaminated soil in a south station of the khurmala oil field in Kurdistan Region. *Iraq. Appl. Sci.-Basel* **2021**, *11(16)*, 7474. https://doi.org/10.3390/app11167474.

[S5] Abdelhalim, R.A.; Selamat, M.R.; Ramli, H. Evaluation of strength properties of oil-contaminated sands upon stabilisation with laterite soil. *Int. J. Pavement Eng.* **2022**, *23(9)*, 2981-2997. https://doi.org/10.1080/10298436.2021.1876875.

[S6] Ahmad, S.; Al-Amoudi, O.S.B.; Mustafa, Y.M.H.; Maslehuddin, M.; Al-Malack, M.H. Stabilization and solidification of oil-contaminated sandy soil using portland cement and supplementary cementitious materials. *J. Mater. Civil Eng.* **2020**, *32(8)*, 04020220. https://doi.org/10.1061/(ASCE)MT.1943-5533.0003169.

[S7] Ahmad, S.; Ba-Naimoon, M.S.M.; Bahraq, A.A.; Al-Amoudi, O.S.B.; Maslehuddin, M.; Al-Malack, M.H. Stabilization/solidification of petroleum oil-contaminated soil using different sabilizers to deliver a pavement subbase material. *Arab. J. Sci. Eng.* **2022**, *47(10)*, 13687-13697. https://doi.org/10.1007/s13369-022-07295-2.
